# Di-n-butyl and di-iso-pentyl phthalates and their mixture increase oxidative stress and embryo-larval malformations in zebrafish (*Danio rerio*)

**DOI:** 10.1590/1678-4685-GMB-2024-0082

**Published:** 2025-06-02

**Authors:** Juliana Roratto Lirola, Tathyana Benetis Piau, Daniela Moraes Leme, Anderson Joel Martino Andrade, Izonete Cristina Guiloski, Helena Cristina Silva de Assis, Cesar Koppe Grisolia, Marta Margarete Cestari

**Affiliations:** 1 Universidade Federal do Paraná, Departamento de Genética, Curitiba, PR, Brazil.; 2 Universidade de Brasília, Instituto de Ciências Biológicas, Departamento de Genética e Morfologia, Brasília, DF, Brazil.; 3 Universidade Federal do Paraná, Departamento de Fisiologia, Curitiba, PR, Brazil.; 4 Universidade Federal do Paraná, Departamento de Farmacologia, Curitiba, PR, Brazil.; 5 Instituto de Pesquisa Pelé Pequeno Príncipe, Curitiba, PR, Brazil.

**Keywords:** Emerging contaminants, zebrafish, ecotoxicology, lipid peroxidation, phthalates

## Abstract

Different phthalate compounds, known as emerging contaminants, such as Di-n-butyl (DBP) and di-iso-pentyl (DiPeP) phthalates, have been found in aquatic environments and are widely used as additives to increase the flexibility of plastics. This study intended to evaluate the toxicity in zebrafish embryos and larvae of DBP and DiPeP, as well as the mixture of the two compounds. Fish embryo toxicity, neurotoxicity, oxidative stress, and genotoxicity studies were carried out in *Danio rerio* (zebrafish) embryos and larvae*.* Fish were exposed to DBP and DiPeP in concentrations ranging from 0.001 to 0.125 mg/L and also a combination of compounds DBP + DiPeP (1:1 MIX). DBP caused mortality and embryo malformations from 0.062 mg/L, DiPeP at 0.125 mg/L and the mixture at 0.031 mg/L, indicating the potential toxicity of these phthalates. Isolated phthalates and in mixture induced neurotoxicity and oxidative stress, at low concentrations in zebrafish larvae. No genotoxicity was found through comet assay in larvae. DBP was more toxic than DiPeP, and the mixture was more toxic than both phthalates when tested in isolation, showing some kind of interaction.

## Introduction

Phthalates or esters of phthalic acids are organic compounds widely used as additives and plasticizers to improve the flexibility, strength, and durability of various commercial products (Staples et al., 1997; Seyoum and Pradhan, 2019). These compounds are not covalently bonded to the plastic matrix, easily reaching the environment (Christen et al., 2012). The increasing use of phthalates has resulted in the presence of these compounds in the soil, air, and water (Souza et al., 2018). Certain isolated phthalates in the aquatic environment have been detected at concentrations of up to 0.05 mg/L, which emphasizes the need for toxicological studies analyzing concentrations similar to those found in the environment (Seyoum and Pradhan, 2019). 

In humans and non-aquatic organisms, phthalates are absorbed orally or after inhalation and dermal contact, and then metabolized and conjugated in the liver into more soluble metabolites for urinary excretion (Souza et al., 2018). Phthalates are potent endocrine disruptors, which can bind to hormone receptors and thus mimic or antagonize the action of the natural ligand hormone or inhibit the biosynthesis of hormones, such as testosterone (Bradlee and Thomas, 2003). The toxic potential combined with the extensive use of phthalates has attracted much attention from the scientific community and regulatory agencies (Souza *et al.,* 2018; Bradlee and Thomas, 2003). 

The toxicity of phthalates is directly related to the side chain length of the ester molecule, ranging from C1 to C13, in which the esters with 3 to 7 carbons in the linear portion of the side chain display higher antiandrogenic activity and reproductive toxicity (Bradlee and Thomas, 2003). Di-n-butyl phthalate (DBP) is a low molecular weight phthalate (C4) classified by the European Agencies as a reproductive toxicant category 1B, *i.e*., presumed to have potential reproductive toxicity for humans, based largely on experimental animal data (Bradlee and Thomas, 2003; Souza et al., 2018). DBP is among the phthalates most used in industry, and its applications include medical products, automotive parts, toys, food packaging and personal hygiene merchandise (Rocha et al., 2017, Xu and Gye, 2018).

Di-iso-pentyl phthalate (DiPeP), also known as diisoamyl phthalate (DiAP), is used in the production of propellants, nitrocellulose explosives, and in PVC products, in addition to other polymers. In Brazil, the local production of DiPeP is favored by the ample availability of isoamyl alcohol, a building block of DiPeP production that is also a product of sugar fermentation in the ethanol production process (Souza et al., 2018). In Brazil, DiPeP use has been banned in personal care products since 2016. However, it is used in several other industrial applications (ANVISA, 2016). In addition, its presence has been identified and quantified in the soil (Ferreira and Morita, 2012), and its metabolites were found in the urine of pregnant Brazilian women (Souza *et al.,* 2018). DiPeP, which presents an intermediate side chain length with five carbons (four linear and a methyl branch), has been reported as a potent antiandrogenic phthalate, displaying higher endocrine disrupting activity than more commonly used phthalate esters like DBP (Souza *et al.,* 2018).

In a realistic situation, humans and animals are continuously exposed, through different routes, to mixtures composed of various phthalates (Zhou et al., 2017). Thus, experimental studies testing mixtures of different chemicals simultaneously might better represent the real exposure scenarios, since the risks of multiple exposures are usually unknown (Christen et al., 2012; Zhou *et al.,* 2017). However, most studies use isolated compounds and investigate their effects on the reproduction of mammals (Zhou *et al.,* 2017; Yang et al., 2018; Seyoum and Pradhan, 2019).

The negative impacts of chemical contamination on survival, growth, and reproductive success should be evaluated in populations and communities (Bradlee and Thomas, 2003). Aquatic organisms are particularly vulnerable to exposure to phthalates; however, the ecotoxicological impact of these compounds in the embryonic development of fish is still unclear (Yang et al., 2018). *Danio rerio* (zebrafish) has been used to study the effects of drugs and environmental contaminants on embryonic development (Felisbino et al., 2023; Santos et al., 2023).

Fish Embryo Toxicity (FET), a test-protocol developed by the Organization for Economic Co-operation and Development - OECD 236 (2013) is routinely used in different studies (Rodrigues de Oliveira et al., 2016; Andrade *et al.,* 2018; Oliveira de Farias et al., 2019). The use of the FET test enables exposure to multiple toxic agents simultaneously, as well as the analysis of biochemical biomarkers, thus providing information at the molecular level (Rivero-Wendt et al., 2016). The addition of the FET, biochemical, and genetic biomarker tests contributes to a better understanding of the toxicity of these compounds (Rivero-Wendt *et al.,* 2016).

Therefore, the present study aims to evaluate the toxicity of the mixture (MIX) of di-n-butyl phthalate (DBP) and di-iso-pentyl phthalate (DiPeP), as well as the isolated effects of each of these emerging contaminants. Toxicity was evaluated in the embryos and larvae of *Danio rerio*, in a multilevel approach analysing different endpoints such as developmental malformations, considering embryo development as the most sensitive bioassay for toxicological evaluations. Neurotoxicity was carried out through acetylcholinesterase (AChE) activity measurement. Biomarkers of oxidative stress were asessed by quantifying glutathione S-transferase (GST) and lipid peroxidation (LPO) measurement. Genotoxicity evaluation was performed through comet assay.

## Material and Methods

The experiments were approved by the Ethics Committee on Animal Use at the Federal University of Parana, Brazil (certificate n. 1138/2017).

### Chemical products and test solutions

Di-n-butyl phthalate (DBP) CAS registry (Chemical Abstract Service) 84-74-2 (99%, Sigma-Aldrich^®^, Darmstadt, Germany) and di-iso-pentyl phthalate (DiPeP), CAS 605-50-5 record (99% Petrom^®^ - Petrochemical Mogi das Cruzes SA, Brazil) were used in the experiments. The stock solutions were prepared at a concentration of 10 mg/L phthalate and used a methanol solvent 0.1% v/v (Chen et al., 2014).

All solutions for exposures were prepared using water from the zebrafish facility, where all water physical-chemical parameters are constantly under control, such as temperature, conductivity, pH, dissolved oxygen, and microbiological control of pathogens. The concentrations were defined after testing for preliminar acute toxicity. The test solutions used were: DBP and DiPeP at concentrations of 0.001, 0.003, 0.007, 0.015, 0.031, 0.062, and 0.125 mg/L, and MIX concentrations of 0.001, 0.003, 0.007, 0.015 and 0.031 mg/L.

Experiment controls were prepared as follows: negative control (NC, facility water), solvent control (SC, 0.1% methanol v/v), and positive control (PC, 4 mg/L 3.4 dichloroaniline [CAS 95-76-1, 99%, Sigma-Aldrich^®^, Darmstadt, Germany]).

Considering the results of preliminary toxicity tests, five lower concentrations of phthalates for the MIX composition were defined. The mixtures were prepared at a ratio DBP: DiPeP (1:1 v/v), ensuring that each phthalate contributes equally to the potential effects at each concentration of the mixture.

### Zebrafish maintenance and embryo collection

Adults were kept in a recirculating zebrafish facility established at the Department of Genetics and Morphology in the University of Brasilia, Brazil (ZebTec - Tecniplast^®^, Italy) in water tanks filtered with activated carbon and reverse osmosis. The fish were kept in a cyclical photoperiod of 12:12h (light: dark); water temperature of 27 ± 1 °C; conductivity 750 ± 50μS/cm; pH 7.0 ± 0.5 and a saturation rate of dissolved oxygen at or above 95%. This maintenance water was used in the internal control, negative control, and for preparation of the test solutions of all toxicity tests. The embryos were obtained immediately after natural mating in the iSpawn system (Tecniplast^®^), and inspected under a stereomicroscope (Stemi 2000 Zeiss, Germany). The unfertilized eggs and those embryos with cleavage irregularities or injuries were discarded.

### Toxicity test in zebrafish embryos

The FET test was performed according to OECD 236 (2013). The experiments were conducted in 24-well plates, with one plate per concentration of each one isolated or mixed phthalate. Each plate contained four wells as an internal control (filled with 2 mL of water from the facility) and 20 wells were filled with 2 mL of the test solution. Fertilized eggs were randomly selected and carefully distributed, with one egg per well. Plates were kept in a climatic chamber at 27 ± 1 °C in a 12 h light-dark cycle. Assays were performed in triplicate, totaling 60 eggs per concentration.

Embryo development was evaluated using a stereomicroscope (Carl Zeiss^®^ Stemi 2000-C) for periods of 24, 48, 72, and 96 hours post-fertilization (hpf). The following lethality parameters according to OECD 236 (OECD-236, 2013) were analyzed: coagulated embryos, lack of somite formation, non-detachment of the tail, and lack of heartbeat. Sublethal parameters were also analyzed: chorion structural deformations, the presence of hemorrhages and edema, yolk sac absorption, skeletal deformities, pigmentation of the eyes and body, reduced larva growth, inflation of the swim bladder, and loss of equilibrium. 

### Biochemical biomarkers: neurotoxicity and oxidative stress

To quantify the activity of acetylcholinesterase (AChE), glutathione S-transferase (GST), and the levels of lipid peroxidation (LPO), pools consisting of 13 larvae were used, containing a sufficient amount of protein for the analyses of the biomarkers.

In each exposure group (NC, SC and DBP, DiPeP, and MIX) 10 pools were prepared for each replica, each pool containing 13 larvae, completing 130 larvae. The larvae were exposed in 6-well cell plates. Each pool was homogenized in a 300 µL of 0.1 M potassium phosphate buffer (pH 7.0) and centrifuged at 10,000xg for 20 min at 4 °C. The supernatant was used to measure the enzyme activities and lipid peroxidation. The total protein concentration was determined using the Bradford method (Bradford, 1976), with bovine serum albumin as the standard.

Acetylcholinesterase activity was measured by the method of Ellman (Ellmann et al., 1961), with the following modifications: in the microplate were added 25 µL of sample, 200 µL DTNB (5.5 - dithiobis-2-nitrobenzoate 0.75 mM) and 50 µL of 7.5 mM acetylthiocholine. The microplate was incubated for 5 min and reading was performed at 405 nm for 5 min every 30 s. Enzymatic activity was expressed in nmol. min^-1^.mg protein^-1^. Glutathione-S-transferase activity was measured according to Keen (Keen et al., 1976) with the following modifications: 20 µL sample was added to a microplate along with a 180 µL reaction solution (GSH 1.5 mM; CDNB the 1.5 mM). The plate was incubated for 5 min and the reading was performed at 340 nm for 5 min every 30 s. Enzymatic activity was expressed in nmol. min^-1^.mg protein^-1^.

The analysis of lipid peroxidation was accomplished by evaluating the concentration of hydroperoxides using the FOX assay (ferrous oxidation/Xylenol Orange Method) (Jiang et al., 1992). The supernatant was resuspended in methanol at 1:2 (v/v) and centrifuged for 5 min 10,000xg at 4 °C. Then, 100 µL of the supernatant was added to microtubes and incubated with a 900 µL reaction solution (100 µM xylenol orange, 25 mM H_2_SO_4_, BHT 4 µM, 250 µM ferrous ammonium sulfate, 90% methanol) for 30 min. The reading was performed at 570 nm and the results were expressed as µmol hydroperoxides. mg. protein^-1^.

### Genetic biomarker

To analyze the genotoxicity in larvae of *Danio rerio*, the alkaline comet assay was used (Singh et al., 1988), modified by Ramsdorf et al. (2009), with the following detailed changes: after the FET test, all the surviving *D. rerio* larvae after 96 hours of exposure were euthanized with tricaine (5%) for 20 min at 20 °C, and placed in microtubes containing 500 µL of fetal bovine serum. They were then stored in a cool dark environment. A pool with larvae from FET test was used to prepare the slides for comet assay (approximately 20 larvae per treatment) for each replica (3 replicas). For each replica, two slides were prepared. 

The larvae belonging to the internal control of the plates were used as the positive control of the comet assay technique and were exposed to ultraviolet light of type C (UV-C, wavelength 320 nm) for 5 min (Kosmehl et al., 2006). The larva pool (n = 20) was disaggregated in a micro-homogenizer (Potter type). Then 120 µL of the larval homogenate taken and mixed in 120 µL of agarose at a low melting point (LMP) and dispensed (100 µL) on each of the two slides. The slides were then placed in a lysis solution for three hours. After this period, they were covered with an alkaline buffer for 25 min and then subjected to electrophoresis (25 V and 300 mA) for 25 min.

The slides were prepared in duplicate, for each replica of the FET test, totaling six slides per treatment. The comet assay was performed in the negative control, positive control, solvent control, DBP, DIPEP, and MIX, based on the same exposure concentrations of the FET.

The slides were stained with 20 µL of ethidium bromide (10 µL/mL). The analysis was performed as a blind test with 100 nucleoids per slide, using an epifluorescence microscope at 400x magnification (Leica^®^, DMLS2 model). The nucleoids were ranked through visual inspection and assigned scores for each class of DNA damage; 0 = no apparent damage, 1 = little damage, damage 2 = medium, 3 = extensive damage, and 4 = maximum damage (Koppen et al., 2017). The score was obtained by the sum of the nucleoid number of comets in each class multiplied by its respective class.

### Statistical analysis

Software BioEstat^®^ 5.0 was used for the analysis. The threshold significance used was 5% (p values <0.05). The Kolmogorov-Smirnov test was used to test the normality of the distribution of variables and to determine the use of parametric or non-parametric tests. The FET test recorded the presence of each of the developmental malformations, and the mean value was then compared among treatments using ANOVA, followed by the Dunnett multiple comparison test. The same procedure was done for the analysis of biomarkers relating to neurotoxicity and oxidative stress. Student’s t-test was used to compare the means for each concentration of isolated phthalates and the mixture. Fold change was used to measure the difference in these means. For each malformation, a general mean was obtained for the isolated phthalates (DBP + DiPeP) and the averages were divided, thus obtaining a ratio that generated the fold change values. To analyze the comet assay data, the Kruskal-Wallis one-way analysis of variance followed by the Student-Newman-Keuls test were used. For EC_50_ determination, the normality test of Shapiro-Wilk was used. As the data showed a normal distribution, (p<0.05), they were analysed by Kruskal-Wallis. The Dunnett test was used to identify possible differences among all exposed groups and the control group. The EC_50_ values were determined by linear regression. It was used the statistical software GraphPad Prisma 8.0.1.

## Results

### Toxicity test in zebrafish embryos

The embryonic mortality values in the negative control (NC) and in the solvent control (SC) groups were less than 10%. The positive control caused a mortality rate higher than 30%, following the quality standards set forth by OECD - 236. The organisms belonging to the NC and SC groups showed normal embryonic development (Kimmel et al., 1995).

**Figure 1:**
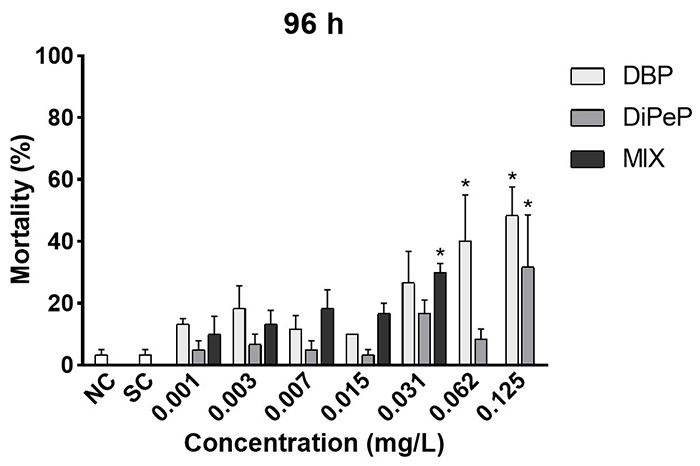
Mortality from FET test in *Danio rerio* embryos and larvae recorded at 96 hours of exposure to di-n-butyl phthalate (DBP), di-iso-pentyl phthalate (DiPeP) and the mixture (MIX). NC (negative control), SC (solvent control - 0.1% methanol v/v), and 0.001; 0.003; 0.007; 0.015; 0.031; 0.062; 0.125 mg/L phthalate. MIX concentrations from 0.001 to 0.031 mg/L. There was mortality at the highest concentrations of MIX. (*) Indicates statistical differences compared to the negative control (p <0.05).

The phthalates induced higher mortality rates of *D. rerio* larvae after 96 hours of exposure in comparison to NC. DBP increased the mortality at concentrations of 0.062 mg/L and 0.125 mg/L, while for DiPeP a higher mortality rate was induced only at the highest concentration (0.125 mg/L). However, the MIX showed a higher toxicity than the two isolated phthalates, thus causing a significant increase in mortality at a concentration of 0.031 mg/L (Figure 1). Based on values of EC_50_ (96 h), it is possible to show that an interaction between the two compounds may be occurring. For DBP - EC_50_ = 0.017 mg/L. For DiPeP - EC_50_ = 0.029. And for MIX (1:1, v/v) EC50 = 0.005 mg/L. Classifying levels of toxicity, these data clearly show MIX > DBP > DiPeP.

**Figure 2:**
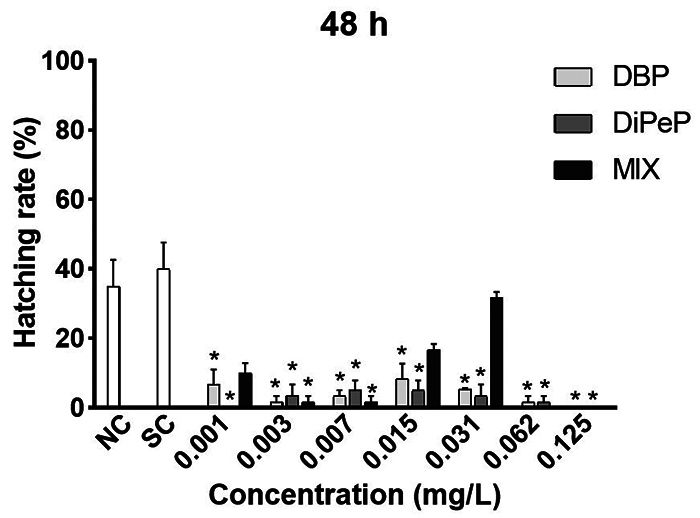
Hatching rate of *Danio rerio* larvae within 48 hours post-fertilization exposed to di-n-butyl phthalate (DBP), di-iso-pentyl phthalate (DiPeP) and the mixture (MIX): NC (negative control); SC (solvent control - 0.1% methanol v/v) and 0.001; 0.003; 0.007; 0.015; 0.031; 0.062; 0.125 mg/L phthalate. MIX concentrations from 0.001 to 0.031 mg/L. (*) Indicates statistical difference compared to the negative control (p <0.05).

The isolated phthalates caused a delay in the hatching of larvae exposed to all the tested concentrations of DBP and DiPeP at 48 hpf. In the mixture, the delay in onset occurred at concentrations of 0.003 and 0.007 mg/L (Figure 2). The hatching rate was normal from 72 hpf.

**Figure 3:**
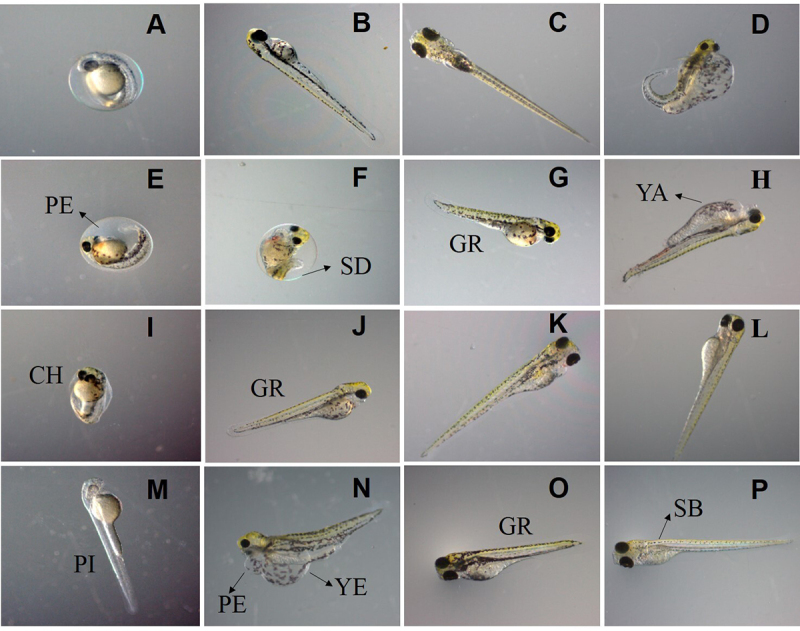
Embryonic development of *Danio rerio* observed in 24, 48, 72, and 96 hours post-fertilization (photo enlargement at 3 x). Embryos and larvae belonging to the negative control group (A, B, and C); Larvae belonging to the positive control group (D); Embryos and larvae exposed to di-n-butyl phthalate (DBP) (E, F, G, and H); Embryos and larvae exposed to di-iso-pentyl phthalate (DiPeP) (I, J, K and L); Larvae exposed to the mix group (MIX) (M, N, O and P). PE = pericardial edema; SD = skeletal deformities; GR = reduced larva growth; YA = non-yolk sac absorption; CH = chorion structural deformations; PI = altered pigmentation; YE = yolk sac edema; SB = uninflated swim bladder.

Throughout the experiment, a variety of malformations were observed through exposure of single phthalates and phthalates as a mixture, such as uninflated swim bladder, non-yolk sac absorption, skeletal deformities, delay in the pigmentation, and pericardial edema (Figure 3). Deformations in the chorion structure were observed only after exposure to 0.125 mg/L DiPeP at 48 hpf (Figure 3). The DBP and MIX did not induce these deformations.

**Figure 4:**
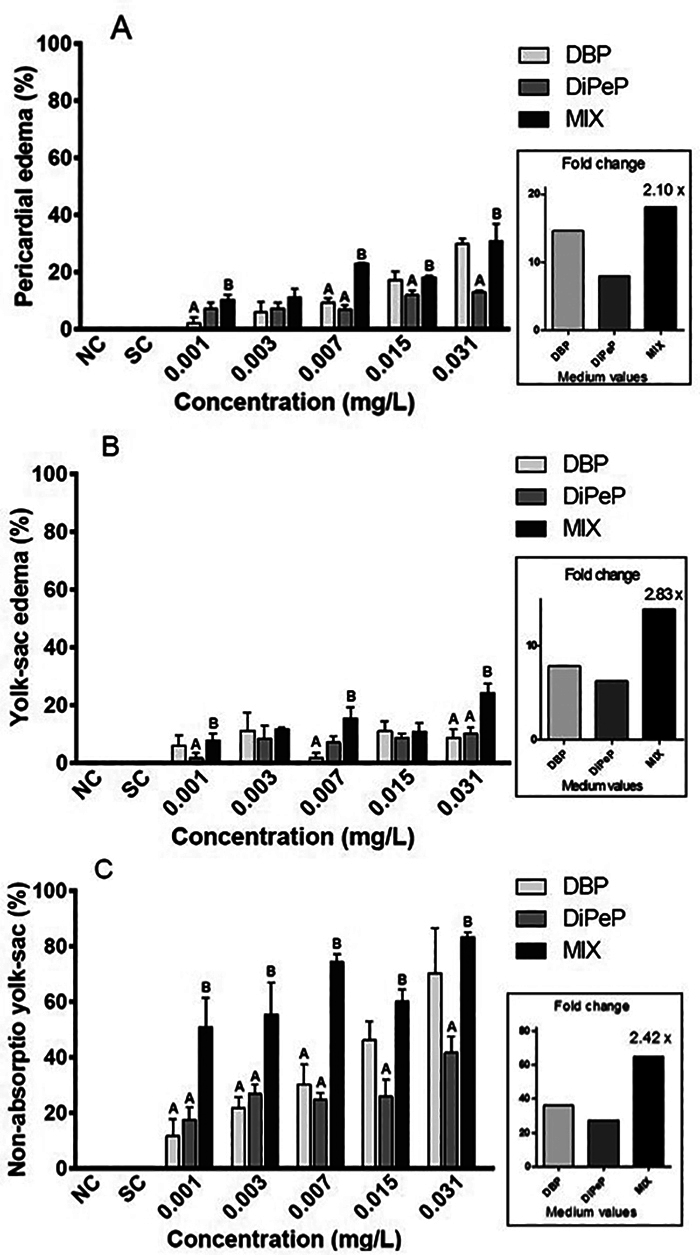
Combined effects of a mixture (MIX) between di-n-butyl phthalate phthalate (DBP) and di-iso-pentyl phthalate (DiPeP) observed by an increase in the average malformations in *Danio rerio* larvae within 96 hours post-fertilization. Analyses were carried out in triplicate. Student’s t-test, with p <0.05. (*) indicates a statistical difference between the DBP and the MIX in the same concentration, and between DiPeP and the MIX in the same concentration (mg/L). To calculate the fold change, a general mean was used, considering all concentrations of isolated phthalates, and a ratio was made with the average of the MIX. Fold change shows the mean increase in damage induced by the MIX when compared to isolated phthalates within each malformation: A - pericardial edema, 2.10 fold increase; B- yolk sac edema, an increase of 2.83 times; C- non-yolk sac absorption, an increase of 2.42 times.

DBP induced pericardial edema (PE) at 0.062 mg/L after 48, 72 and 96 hpf. DiPeP caused PE at 0.125 mg/L after 48 and 72 hpf. Pericardial edema was observed in the MIX from 0.015 mg/L at 48 hpf, and from 0.001 mg/L at 72 and 96 hpf, evidencing that the larvae exposed to all mixture concentrations showed malformations. The PE was more evident when exposures occurred to phthalate mixtures. On average, the pericardial edema occurred at 2.10-fold increases in embryos and larvae exposed to the MIX when compared to those exposed to isolated phthalates (Figure 4A).

With the exposure to DBP at 0.125 mg/L, yolk sac edema (YE) was observed at 24, 48, 72, and 96 hpf. The exposure to DiPeP caused YE only 24 hpf in *D. rerio*. The MIX induced YE with 0.031 mg/L at 24, 48, and 72 hpf, and at 96 hpf, YE was observed in the 0.003 mg/L MIX. The YE malformations demonstrated combined effects in the MIX, showing a 2.83-fold increase when compared to the isolated phthalates (Figure 4B).

Non-yolk sac absorption (YA) was the most common malformation found with the exposure to isolated phthalates and the mixed group. YA were observed at 24 and 48 hours of exposure to the 0.125 mg/L of DBP at 72 hpf with the concentration of 0.031 mg/L. Additionally, at 96 hpf, larvae exposed to concentrations as low as 0.015 mg/L DBP also exhibited YA. At 48 hpf, the concentration of 0.125 mg/L of DiPeP induced YA and, after 72 hpf, YA was observed in larvae exposed to 0.031, 0.062, and 0.125 mg/L of DiPeP. In the MIX, YA defects were observed at 72 hpf and 96 hpf at all tested concentrations. The combined effects of MIX were also observed for YA, at a 2.42-fold increase over isolated phthalates (Figure 4C).

The reduction in the larval growth (GR) was observed at 96 hours of exposure to 0.125 mg/L of DBP, and 72 hours of exposure to 0.031 mg/L of MIX. GR was not observed with the exposure to DiPeP. In addition to a decrease in body size, skeletal deformations were caused by BPD at 0.031, 0.062, and 0.125 mg/L, and MIX at 0.015 and 0.031 mg/L.

Altered pigmentation (PI) occurred in larvae exposed to the highest concentrations of DBP (0.125 mg/L) and DiPeP (0.125 mg/L) at 48, 72 and 96 hpf. The MIX caused less intense pigmentation in the concentration of 0.031 mg/L at 96 hpf. An uninflated swim bladder was observed at 96 hours of exposure to 0.031 mg/L, 0.062 mg/L, and 0.125 mg/L of DBP; 0.125 mg/L of DiPeP and 0.003 mg/L, 0.007 mg/L, 0.015 mg/L and 0.031 mg/L of the MIX concentration. Behavioral changes assessed by loss of equilibrium during swimming were observed in the exposed larvae only at 96 hpf: in the DBP concentration of 0.031 mg/L; 0.125 mg/L of DiPeP, and from 0.003 mg/L of the MIX.

### Biochemical biomarkers: Neurotoxicity and oxidative stress

**Figure 5:**
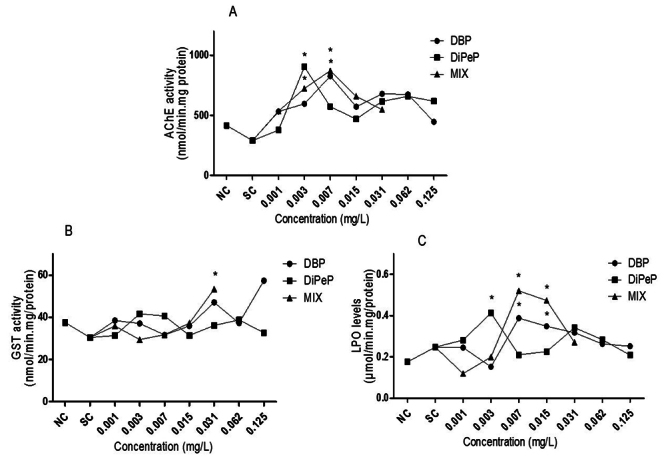
Biochemical biomarkers in *Danio rerio* larvae. Enzyme activity A- Acetylcholinesterase (AChE); B- Glutathione S-transferase (GST) and C- Lipid peroxidation (LPO), showing the pattern of nonmonotonic dose-response. AChE: nmol/mg.protein/min; GST: nmol/min.mg/protein; LPO: µmol/min.mg.protein. Comparison between treatments: negative control (NC), SC (solvent control - 0.1% methanol v/v) and the groups exposed to di-n-butyl phthalate, to di-iso-pentyl phthalate (0.001; 0.003; 0.007; 0.015; 0.031; 0.062 and 0.125 mg/L) and the groups exposed to the mixture of DiPeP + DBP (0.001; 0.003; 0.007; 0.015; 0.031 mg/L). Kruskal-Wallis followed by Dunn test, p <0.05 considered significant. * indicates a significant difference from the negative control.

Increased AChE activity was observed in the exposed larvae at 0.007 mg/L of DBP, 0.003 mg/L of DiPeP, and at 0.003 mg/L and 0.007 mg/L in the MIX (Figure 5A) compared to NC (P<0.05). This demonstrates that the observed effects in biochemical biomarkers show non-monotonic dose-response.

GST enzyme activity was significantly higher in the larvae exposed to 0.031 mg/L of the MIX (Figure 5B), while isolated DBP and DiPeP did not alter the activity of this enzyme. There was an increase in the levels of LPO at 0.007 and 0.015 mg/L of DBP, at 0.003 mg/L of DiPeP, and 0.007 mg/L and 0.015 mg/L in the MIX (Figure 5C). The effect on LPO also appeared at low concentrations, again demonstrating that the standard concentration-response is non-monotonic. The larvae used to evaluate the biochemical markers were the same as those used in the FET test.

### Comet assay

No genotoxicity was observed in the larvae exposed to phthalates, in any of the tested concentrations of DBP, DiPeP, and MIX in 96 hpf.

## Discussion

The present study evaluated the toxicity of different concentrations of two phthalates, and their mixture, which proved to be more toxic than the isolated phthalates. It is important to consider that both humans and other animals are daily exposed to a mixture of phthalates (Christen et al., 2012; Zhou et al., 2017; Souza et al., 2018). The mixtures tested in this study, even at lower concentrations than those usually found in the aquatic environment (0.05 mg/L) (Seyoum and Pradhan, 2019), caused mortality in zebrafish embryos. 

The mortality observed in larvae exposed to DBP, DiPeP, and the MIX was significantly higher than the negative control at 96 hpf. According to Learmonth and Carvalho (2015), newly hatched larvae, at 72 and 96 hpf, showed greater sensitivity than the embryos. As the gills have already formed at 96 hpf, the uptake is more efficient about xenobiotics by the larvae of *D. rerio* and thus may increase the mortality rates, as observed in the present study. In addition, the chorion membrane should protect the embryo from complex molecules. This is probably a reason for the higher toxicity observed after hatching, even though some molecules can cross the chorion pores because phthalates are lipophilic.

In this study, various sublethal endpoints, such as delay in the development, pigmentation delay, embryo tail detachment delay, uninflated swim bladder, and non-reabsorption of the yolk sac were changed following exposure to phthalates, which may be due to the potential endocrine-disrupting activities of these compounds. Considering the assessment of these parameters, the FET test is more sensitive for estimating the acute toxicity of the tested xenobiotics. Many of the sublethal parameters observed respond to neurotoxic compounds, and may have implications for long-term exposures (Stelzer et al., 2018).

This study showed a delay in the hatching of embryos exposed to all DBP and DiPeP concentrations and for MIX at a lower concentration. Hatching is the result of osmotic and enzymatic processes, and embryo movements (Hallare et al., 2005; Oliveira de Farias et al., 2019). The effect of these toxic substances on the hatching process can be derived from dysfunction, or even inhibition of the chorionase enzyme, thus preventing the breaking of the chorion and thereby delaying the hatching of larvae (Hallare *et al.,* 2005).

Some substances can cross the chorion barrier and reach the embryo (Jezierska et al., 2009). After 26 hpf, the embryo enters the pre-hatching stage, and the chorion becomes more permeable due to an increase in proteolytic enzyme activity. This increase in permeability appears to be related to the emergence of deformities in the chorion, which were observed only at 48 hpf with DiPeP in the present study. Interestingly, the chorion barrier is more efficient for chemical compounds with higher molecular weights (Dang et al., 2017). Probably, DiPeP had more difficulty in crossing the chorion barrier in comparison with DBP, because DiPeP has five carbon atoms (C5) in its ester side chain while DBP has only four (C4).

Pericardial edema (PE) is characterized by an increase in the accumulation of pericardial fluid, which can lead to serious changes in cardiovascular development and cause lasting effects on organisms (Hallare et al., 2005). The occurrence of pericardial edema coupled with yolk sac edema (YE), which was observed in the present study, is known as blue sac syndrome, a non-infectious disease caused by exposure to chemicals. The YE is an important toxicological endpoint and because of its role in the early stages of development of *D. rerio*, it may cause coagulation and embryonic death (Rodrigues de Oliveira et al., 2016).

Yolk sac edema is associated with non-yolk sac absorption (YA). The yolk consists of vitamins, proteins, and lipids, serving as a nutrient reserve until the larvae begin the feeding period. As this is a critical period, any abnormality in the consumption of yolk can affect the entire larval development and survival (Ortiz-Zarragoitia et al., 2006; Lourenço et al., 2017). In this study, the YA appeared in embryos and larvae exposed to phthalates in a manner dependent on the concentration and exposure time. It is known that certain xenobiotics, including pharmaceuticals, DBP, and metals, can cause YA in fish embryos (Hallare et al., 2005; Jezierska et al., 2009; Lourenço *et al.,* 2017; Oliveira de Farias et al., 2019).

This delay in yolk absorption can lead to a reduction in metabolic activity, and may affect zebrafish larval growth (Lourenço et al., 2017). Thus, we observed that the larvae had a reduction in body size (GR) when exposed to DBP and the MIX. The mechanisms of action that cause growth inhibition after phthalate exposures are still not completely known. However, studies suggest that phthalates may interact with growth hormones and delay development (Yang et al., 2018). GR is an important variable for toxicity assessment, and the reduction in larval body size observed in the present study may be linked to persistent growth deficits throughout the development and consequently long-term effects up to adulthood (Yang *et al.,* 2018).

The pigmentation in zebrafish embryos begins at 24 hpf with the emergence of melanophores, and it is important for fish survival, by helping to camouflage and avoid predators (Kimmel et al., 1995). Exposure to chemicals can lead to changes in hormonal control and cause abnormalities in the production of melanin, as may have happened in the present work with exposure to phthalates (Kim *et al.,* 2013).

The swim bladder is responsible for adjusting the body density and fluctuation in fish (Yang et al., 2018). The inflation of the swim bladder occurs at 72 hpf in *Danio rerio* (Kimmel et al., 1995). Following exposures to high concentrations of DBP and MIX, a delay in the development of the organ was observed. Defects in this organ impair vital functions of the animal such as swimming, buoyancy, and food search, which in turn may affect the equilibrium and the survival of the animals (Rodrigues de Oliveira et al., 2016).

Swimming is essential for the survival of fish, and changes in this pattern of larvae are considered behavioral changes that may affect the search for food and reproduction. *Danio rerio* larvae are known as a reliable model for studying the behavioral effects caused by exposure to various pharmaceutical drugs, including mixtures of chemicals (Andrade *et al.,* 2018). The loss of equilibrium occurs when swimming larvae at 96 hpf are located at the bottom of the microplate well (Oliveira de Farias et al., 2019). A previous study had shown that exposure to 0.6 mg/L of di-ethyl-hexyl phthalate (DEHP), besides leading to a loss of equilibrium, caused the slowing of movements in the larvae (Staples et al., 1997). In this study, the loss of equilibrium in the larvae exposed to DBP, DiPeP, and the lowest concentrations of the MIX caused the larvae to stay at the bottom of the well in the microplate.

There are few studies analyzing the ecotoxicological effects of phthalates in aquatic animals, particularly in the early stages of fish development (Yang et al., 2018; Oliveira de Farias et al., 2019; Seyoum and Pradhan, 2019). Our results from the FET test, for lethal and sublethal parameters, indicated that the mixture becomes more toxic, signaling that some kind of interaction between the two compounds may be occurring. This is in contrast to the reported endocrine toxicity in rodents, which display higher susceptibility to DiPeP than DBP for the inhibition of testosterone production by fetal rat testes (Souza et al., 2018). These observations support the notion that the susceptibility to environmental chemicals in a certain species cannot fully predict toxicity to other organisms.

This work was the first to describe the ecotoxicological effects of a mixture of DBP and DiPeP in *Danio rerio* at environmentally relevant concentrations. Studies assessing the toxicity of mixtures of compounds are of great relevance since the experimental conditions are more similar to an actual environmental setting, in which animals are exposed daily to a mixture of phthalates and other xenobiotics (Christen et al., 2012; Zhou et al., 2017; Souza et al., 2018). It is worth considering that each mixture will have a different combination effect, but in general, we can deduce that mixtures between phthalates can be more toxic than isolated compounds (Christen *et al.,* 2012).

Generally, exposure to low concentrations of xenobiotics initially results in subtle changes at the molecular level, posing difficulties in the identification and characterization of the exposure effects (Oliveira de Farias et al., 2019). In this study, it was found that changes in the biomarkers of neurotoxicity and lipid peroxidation appeared in low concentrations, demonstrating that these biomarkers may be the first line of defense when exposure to phthalates occurs. The appearance of non-monotonic concentration-response curves or an inverted U curve has already been described for endocrine disrupters such as phthalates, with different doses producing different effects (Vandenberg et al., 2012).

AChE is an important enzyme in neurotransmission and is a key biomarker of the neurotoxicity of environmental compounds (Rivero-Wendt et al., 2016). In association with the FET, it can assist in identifying test compounds that alter neurological development in fish (Kaviraj and Gupta, 2014). This study showed an increase in AChE activity with exposure to DBP, DiPeP, and MIX; however, the mechanism of action of phthalates that caused this increase has not yet been described. This increase in enzyme activity may result in cell apoptosis or cause changes in the autonomic nervous system, and consequently affect behavioral responses, like the loss of equilibrium of zebrafish larvae exposed to DBP, DiPeP, and MIX (Hinojosa et al., 2019).

Phthalates can have multiple sites of interaction and interfere with different biological processes that can result in a wide range of effects, including androgen deficiency, altered cell cycles, changes in metabolic functions, neurotoxicity, behavioral changes, and oxidative stress (Mankidy et al., 2013; Rocha et al., 2017; Yang et al., 2018; Seyoum and Pradhan, 2019). It has already been described that exposure to DEHP and di-ethyl phthalate (DEP) can cause toxicity mediated by oxidative stress in fish embryos (Yang *et al.,* 2018; Seyoum and Pradhan, 2019). Oxidative stress occurs when there is an imbalance in the formation of reactive oxygen species (ROS) and the organism’s ability to eliminate them (Rocha *et al.,* 2017).

The main biological role of antioxidant enzymes such as glutathione S-transferase (GST) is to protect cells from harmful effects caused by ROS and other cell-toxic molecules. An increased GST activity in the liver of birds exposed to DEHP was reported by Zhang et al. (2019). The same results were observed in this study, in which we found increases in GST activity, but only when embryos were exposed to the highest concentrations of MIX, which may indicate a counter-regulatory response to the harmful effects of the phthalate association. 

This demonstrates once again that the MIX is more toxic than isolated phthalates. These results demonstrate that exposures to phthalates can modulate this antioxidant defense system to prevent damage to macromolecules such as proteins, lipids, and DNA (Yang et al., 2018; Zhang et al., 2019). ROS are highly reactive compounds and their accumulation can cause damage to macromolecules, such as lipid peroxidation (LPO). The induction of LPO in zebrafish embryos exposed to phthalate indicates that LPO is a sensitive exposure biomarker (Zhang *et al.,* 2019). Other studies confirm our results, in which phthalate exposures increased LPO levels in *Pimephales promelas* embryos exposed to DEP (10 mg/L) and DEHP (1 mg/L), and in *Xenopus laevis* embryos exposed to DBP (1.1 mg/L) (Mankidy et al., 2013; Xu and Gye, 2018). 

The ecotoxicological impact of phthalate exposures during the early stages of fish life at environmentally relevant concentrations remains uncertain (Yang et al., 2018). Considering that the larval stage is a period of higher sensitivity to the action of these xenobiotics, and normal development requires that critical events occur properly, the observation of malformations in embryos emphasizes the importance of understanding these compounds’ toxicity mechanisms (Zhou et al., 2017).

It is important to take into account that different phthalates have different mechanisms of toxicity, which may result in heterogeneous responses to isolated compounds or mixtures according to the experimental settings, life stage, and animal model (Mankidy et al., 2013). In this study, mortality and developmental abnormalities were observed in embryos and larvae exposed to the two isolated phthalates and their association at environmentally relevant concentrations. Some malformations appeared in DBP and DiPeP when tested in isolation, while when tested as a mixture the range of malformations was greater. It was evident that the MIX was more toxic, thus causing these negative effects at concentrations below the isolated phthalates. For genotoxic evaluation, only the comet assay was performed. Since this result was negative, the micronucleus test as next the step previously programmed was not performed, once the comet assay was more sensitive.

## Conclusions

The tested phthalates showed differences in toxicity, where DBP was more toxic than DiPeP in zebrafish larvae, which is in contrast to rodent toxicity data. The mixture was more toxic than the two isolated phthalates concerning the mortality rate, but also using the sublethal FET test parameters and assessing the activity of GST. The evaluation of isolated compounds, when done independently, can lead to an underestimation of the adverse effects of phthalates. An interactive effect between the two compounds should be better investigated since they are simultaneously found in aquatic environments.
